# Serum adiponectin is positively associated with lung function in young adults, independent of obesity: The CARDIA study

**DOI:** 10.1186/1465-9921-11-176

**Published:** 2010-12-09

**Authors:** Bharat Thyagarajan, David R Jacobs, Lewis J Smith, Ravi Kalhan, Myron D Gross, Akshay Sood

**Affiliations:** 1 Department of Laboratory Medicine and Pathology, Medical School, University of Minnesota Minneapolis, MN 55455, USA; 2 Division of Epidemiology and Community Health, School of Public Health, University of Minnesota, Minneapolis, MN 55454, USA; 3 Department of Nutrition, University of Oslo, Oslo, Norway; 4 Asthma-COPD Program, Division of Pulmonary and Critical Care, Northwestern University Feinberg School of Medicine, Chicago, IL 60611, USA; 5 Division of Pulmonary and Critical Care Medicine, University of New Mexico, Albuquerque, NM 87131, USA

## Abstract

**Rationale:**

Adipose tissue produces adiponectin, an anti-inflammatory protein. Adiponectin deficiency in mice is associated with abnormal post-natal alveolar development.

**Objective:**

We hypothesized that lower serum adiponectin concentrations are associated with lower lung function in humans, independent of obesity. We explored mediation of this association by insulin resistance and systemic inflammation.

**Methods and Measurements:**

Spirometry testing was conducted at years 10 and 20 follow-up evaluation visits in 2,056 eligible young adult participants in the Coronary Artery Risk Development in Young Adults (CARDIA) study. Body mass index, serum adiponectin, serum C-reactive protein (a marker of systemic inflammation), and insulin resistance were assessed at year 15.

**Main Results:**

After controlling for body mass index, years 10 and 20 forced vital capacity (FVC) were 81 ml and 82 ml lower respectively (p = 0.004 and 0.01 respectively) in the lowest *vs*. highest adiponectin quartiles. Similarly, years 10 and 20 forced expiratory volume in one second (FEV_1_) were 50 ml and 38 ml lower (p = 0.01 and 0.09, respectively) in the lowest *vs*. highest adiponectin quartiles. These associations were no longer significant after adjustment for insulin resistance and C-reactive protein. Serum adiponectin was not associated with FEV_1_/FVC or peak FEV_1_.

**Conclusions:**

Independent of obesity, lower serum adiponectin concentrations are associated with lower lung function. The attenuation of this association after adjustment for insulin resistance and systemic inflammation suggests that these covariates are on a causal pathway linking adiponectin and lung function.

## Introduction

Adipose tissue produces adipokines, proteins that regulate inflammation and metabolism in an autocrine, paracrine, and systemic manner [1]. Adiponectin is an adipokine associated with systemic anti-inflammatory effects and insulin sensitization effects [1]. Serum adiponectin concentrations are reduced in obesity [2,3]. Adiponectin and all of the known receptors for adiponectin (AdipoR1, AdipoR2, T-cadherin and calreticulin) are expressed on multiple cell types in the lung [4-7]. Adiponectin is also transported from blood into the alveolar lining fluid via the T-cadherin molecule on the endothelium [5].

Various disease states associated with lower serum adiponectin concentrations (such as obesity, asthma, systemic inflammation, and diabetes mellitus [2,3,8,9]) are associated with reduced lung function [10-14]. It is therefore possible that lower serum concentrations of adiponectin may be associated with decreased lung function in humans. This hypothesis is supported by a recent study in normal-weight mice with genetic deficiency of systemic adiponectin. These mice demonstrated local (lung) adiponectin deficiency, increased systemic and local inflammation, and "alveolar simplification and/or enlargement due to abnormal post-natal alveolar development" [15].

In this study, we evaluated the association between serum concentration of adiponectin and lung function in the prospective Coronary Artery Risk Development in Young Adults (CARDIA) study. We hypothesized that lower serum concentrations of adiponectin measured at year 15 after enrollment in the CARDIA study are associated with lower lung function at years 10 and 20 and ten year decline in lung function. We also hypothesized that this effect of serum adiponectin concentrations on lung function is independent of obesity and might be mediated by its systemic anti-inflammatory and insulin sensitization effects.

## Methods

In the CARDIA study, 5,115 participants aged 18-30 years were recruited for the baseline (year 0) examination in 1985-86, including approximately equal numbers who were black and white, men and women, and ≤ twelve-years of education and > twelve-years of education. Subsequently, 3,950 participants (77%) were followed up in 1995-96 (year 10); 3,672 (72%) in 2000-2001 (year 15); and 3,549 (69%) in 2005-2006 (year 20) examinations. The detailed methods, instruments and quality control procedures for the CARDIA study have been previously described [10,16]. The CARDIA study is reviewed annually by the internal review boards at each participating institution, and participants sign a new informed consent form at every examination. Demographic characteristics, lifestyle habits (e.g. cigarette smoking), physical activity, and medical history were collected by self-report. The diagnosis of asthma was made if the subject at any of the study visits self-reported a doctor or nurse diagnosis of asthma and/or reported taking asthma medications (usually based on examination of medicine containers). Spirometry was performed using a Collins Survey 8-liter water sealed spirometer and an Eagle II Microprocessor (Warren E. Collins, Inc., Braintree, MA) at year 10 examination and a dry rolling-seal OMI spirometer (Viasys Corp, Loma Linda, CA) at year 20 examination, adhering to the American Thoracic Society guidelines. A comparability study performed on 25 volunteers at the LDS Hospital (Salt Lake City, UT) demonstrated excellent consistency between the old and new machines; the average difference between the Collins Survey and OMI spirometer was 6 ml for FVC and 21 ml for FEV_1_. The homoeostasis model assessment (HOMA) for estimating insulin resistance was calculated as serum glucose (mmol/L)×serum insulin (mU/L)/22·5 [17]. Serum C reactive protein (CRP) was measured using a high sensitivity new enzyme-linked immunosorbent assay method at the Department of Pathology, University of Vermont, Burlington, VT, USA [18].

After excluding participants who were pregnant during years 10, 15 or year 20 examination (n = 16), asthma diagnosis during the 20 years of CARDIA follow-up (n = 408), and those with missing values for either serum adiponectin (n = 423), year 20 lung function (n = 119), year 10 lung function (n = 447) or covariates (n = 80), 2,056 participants were included in this study.

Overnight fasting blood samples were collected, processed within 90 minutes of blood collection and stored at -70°C. Total adiponectin was measured in serum by radioimmunoassay at Linco Research, Inc. (St. Louis, MO) using a polyclonal antibody raised in a rabbit with an effective range of 0.2 to 40 mg/L [8]. The correlation between adiponectin concentrations measured in 407 blinded duplicate samples was 0.91 and the coefficient of variation (CV) for the adiponectin assay was 17% (including laboratory measurement error, variation in specimen handling and freezing and labeling errors). A previous study demonstrated that serum adiponectin showed little circadian variability and limited within-person variation over time [19].

## Statistical Analysis

We used linear regression (PROC GLM) in SAS, version 9.1 (Cary, NC) to evaluate the associations of year 15 adiponectin concentrations with lung function at years 10 and 20. Quartiles of year 15 adiponectin concentrations (independent variable) were used to predict forced vital capacity (FVC), forced expiratory volume in one-second (FEV_1_) and FEV_1_/FVC ratio at years 10 and 20 (dependent variables). In addition, the ten-year change (year 20 - year 10) in FVC, FEV_1 _and FEV_1_/FVC was predicted, using year 15 adiponectin quartiles. Of note, serum adiponectin measurements at years 10 and 20 or conversely, spirometric measurement at year 15 were not available in the CARDIA study. These associations were adjusted for race, sex, study center, height, height^2^, age, age^2^, amount of self-reported physical activity, body mass index (BMI) and smoking status (never, former, current), all at year 15 (model 1). These covariates were selected because of their association with lung function or adiponectin. Since age and height were not linearly associated with lung function, age^2 ^and height^2 ^were included in the model to more completely adjust the effect of age and height on lung function. In addition, all analyses were performed with adjustment for measures of insulin resistance (as estimated by HOMA and systemic inflammation (CRP) (model 2). All these analyses (models 1 and 2) were also performed with adiponectin as a continuous variable. Adiponectin, HOMA and CRP were (natural) logarithmically transformed (ln) due to their skewed distribution. There was no evidence for collinearity among the covariates (except age-age^2 ^and height-height^2^) used in the model. We also analyzed the association between year 15 serum adiponectin concentrations (independent variable) and peak lung function (FVC, FEV_1 _and FEV_1_/FVC) in early adulthood (dependent variable) using models similar to those described above in which all the covariates were measured at year 5. Peak lung function was defined as the maximum lung function measurement at any of the three initial CARDIA visits (years 0, 2 or 5 visits).

## Results

### Participant characteristics at Year 15 examination

At year 15, participants in the highest serum adiponectin quartile were more likely to be white, women, have higher educational attainment, and be former smokers (and less likely to be current smokers), as compared to those in the lowest adiponectin quartile (p < 0.001; Table 1). Consistent with previous reports, BMI, insulin resistance (HOMA) and systemic inflammation (serum CRP levels) were lower in the highest *vs*. lowest adiponectin quartile (p < 0.001; Table 1) [2,3,8]. Body mass index, HOMA and CRP were also significantly correlated with each other (r_BMI, CRP _= 0.53, r_BMI, HOMA _= 0.60 and r_HOMA, CRP _= 0.41, all p < 0.0001).

**Table 1:** Clinical characteristics according to quartiles of year 15 serum adiponectin concentrations; CARDIA "calendar years".

Year 15 adiponectin quartiles (Q)	Q1 (n = 565)	Q2 (n = 449)	Q3 (n = 519)	Q4 (n = 523)	p value
Age (years)	40.2 (3.7)	40.2 (3.6)	40.2 (3.6)	40.5 (3.5)	0.51
Race (% blacks)	62	46	36	27	<0.001
Sex (% men)	70	53	41	22	<0.001
Education level (completed high school or lower) (%)	26	20	17	17	<0.001
Smoking Status					0.003
Never smoker, %	65	65	65	64	
Former smoker, %	14	14	19	21	
Current smoker, %	21	20	16	15	
Body Mass Index (kg/m2)	31.0 (6.1)	29.5 (6.9)	27.9 (6.2)	25.3 (5.2)	<0.001
Physical Activity score (exercise units)	355 (301)	347 (288)	352 (286)	369 (275)	0.66
Serum C-reactive protein (g/ml)	3.9 (5.1)	3.0 (3.8)	2.8 (6.0)	1.9 (3.2)	<0.001
ln (Homeostasis Model Assessment, HOMA units)	1.5 (0.5)	1.3 (0.4)	1.2 (0.4)	1.1 (0.4)	<0.001
FVC at year 10 (ml)	4464 (1027)	4395 (1064)	4381 (1046)	4204 (942)	<0.001
FEV_1 _at year 10 (ml)	3568 (775)	3507 (813)	3510 (793)	3371 (696)	<0.001
FEV_1_/FVC at year 10 (%)	80.4 (5.5)	80.3 (5.7)	80.5 (5.5)	80.7 (6.1)	0.65

Note 1: All variables were measured at year 15 except Forced vital capacity (FVC), Forced Expiratory Volume in 1 second (FEV_1_) and FEV_1_/FVC that were measured at year 10 and are expressed as mean (standard deviation) or percent.

Note 2: Quartile cutpoints for serum adiponectin concentrations are ≤6.99, 7.00-9.59, 9.60-14.39, and ≥ 14.40 mg/l.

### Year 15 serum adiponectin concentrations were positively associated with Year 10 FVC and FEV_1_

Year 10 FVC was 81 ml lower in the lowest *vs*. highest adiponectin quartile (p for trend = 0.005; Table 2, model 1). Similarly, year 10 FEV_1 _was 50 ml lower in the lowest vs. highest adiponectin quartile (p for trend = 0.01; Table 2, model 1). Adjustment for either year 15 waist circumference or year 10 BMI instead of year 15 BMI in this model showed results very similar to that observed after adjustment for year 15 BMI (data not shown). However, after additional adjustment for insulin resistance and systemic inflammation, year 10 FVC and FEV_1 _were not associated with adiponectin (Table 2, model 2), suggesting that these are possible mechanisms for the adiponectin-lung function association. Year 10 FEV_1_/FVC was not associated with adiponectin in any of the models (Table 2).

**Table 2:** Association between year 15 serum adiponectin concentrations and year 10 lung function; CARDIA "calendar years".

*Year 15 adiponectin quartiles (Q)*	YEAR 10 LUNG FUNCTION PARAMETERS (95% CI)	
	MODEL 1 (n = 2,056)	MODEL 2 (n = 2,056)
	**Year 10 FVC (ml) ± SE**	
Q1	4298 ± 23	4327 ± 24
Q2	4353 ± 24	4357 ± 24
Q3	4420 ± 22	4409 ± 22
Q4	4380 ± 25	4357 ± 25
Q1 minus Q4	-81 (-153, -9)	-30 (-103, 43)
Slope of ln(adiponectin)*	-82 (-140, -23)	-35 (-94, 25)
	**p for trend = 0.005**	**p for trend = 0.25**
		
	**Year 10 FEV_1 _(ml) ± SE**	
Q1	3448 ± 19	3469 ± 20
Q2	3476 ± 20	3479 ± 20
Q3	3539 ± 18	3531 ± 18
Q4	3498 ± 20	3481 ± 20
Q1 minus Q4	-50 (-109, 9)	-12 (-72, 48)
Slope of ln(adiponectin)*	-61 (-109, -13)	-27 (-76, 23)
	**p for trend = 0.01**	**p for trend = 0.28**
		
	**Year 10 FEV_1_/FVC (%) ± SE**	
Q1	80.7 ± 0.2	80.6 ± 0.3
Q2	80.3 ± 0.3	80.3 ± 0.3
Q3	80.5 ± 0.2	80.5 ± 0.2
Q4	80.3 ± 0.3	80.4 ± 0.3
Q1 minus Q4	0.4 (-0.4, 1.1)	0.3 (-0.5, 1.1)
Slope of ln(adiponectin)*	0.11 (-0.51, 0.73)	0.03 (-0.61, 0.68)
	**p for trend = 0.73**	**p for trend = 0.92**

Note 1: Model 1 was adjusted for race, sex, center, age, age^2^, amount of self-reported physical activity, body mass index (BMI) and smoking status (never, former, current) all at year 15 and height, height^2^at year 0. Model 2 was adjusted for all covariates in model 1 and for ln(insulin resistance in HOMA units) and ln(CRP) at year 15 examination.

Note 2: Quartile cutpoints for serum adiponectin concentrations are ≤6.99, 7.00-9.59, 9.60-14.39, and ≥ 14.40 mg/l. Mean ± standard deviation of ln(adiponectin) = 2.21 ± 0.62 mg/l

* Unit of slope was selected to match the adiponectin difference across Q1 to Q4. It was ml/(median level of adiponectin in quartile 1 (4.8 mg/l) - median level of adiponectin in quartile 4 (18.0 mg/l)).

### Year 15 serum adiponectin concentrations were positively associated with Year 20 FVC and FEV_1_

Year 20 FVC was 82 ml lower in the lowest *vs*. highest adiponectin quartile (p for trend = 0.01; Table 3, model 1). Similarly, year 20 FEV_1 _was 38 ml lower in the lowest vs. highest adiponectin quartile, this difference showed a trend towards statistical significance (p for trend = 0.09; Table 3, model 1). Adjustment for either year 15 waist circumference or year 20 BMI instead of year 15 BMI in this model showed results very similar to that observed after adjustment for year 15 BMI (data not shown). However, after additional adjustment for insulin resistance and systemic inflammation, year 20 FVC and FEV_1 _were not associated with adiponectin (Table 3, model 2). Year 20 FEV_1_/FVC was not associated with adiponectin concentrations (Table 3).

**Table 3:** Association between year 15 serum adiponectin concentrations and year 20 lung function; CARDIA "calendar years".

*Year 15 adiponectin quartiles (Q)*	YEAR 20 LUNG FUNCTION PARAMETERS (95% CI)	
	MODEL 1 (n = 2,056)	MODEL 2 (n = 2,056)
	**Year 20 FVC (ml) ± SE**	
Q1	3916 ± 24	3953 ± 24
Q2	3968 ± 25	3973 ± 25
Q3	4032 ± 23	4017 ± 23
Q4	3997 ± 26	3967 ± 25
Q1 minus Q4	-82 (-156, -7)	-15 (-90, 61)
Slope of ln(adiponectin)*	-75 (-135, -14)	-13 (-75, 49)
	**p for trend = 0.01**	**p for trend = 0.67**
		
	**Year 20 FEV_1 _(ml) ± SE**	
Q1	3101 ± 20	3126 ± 20
Q2	3124 ± 21	3128 ± 20
Q3	3171 ± 19	3162 ± 19
Q4	3138 ± 21	3117 ± 21
Q1 minus Q4	-38 (-99, 24)	9 (-53, 72)
Slope of ln(adiponectin)*	-42 (-92, 8)	1 (-50, 52)
	**p for trend = 0.09**	**p for trend = 0.97**
		
	**Year 20 FEV1/FVC (%) ± SE**	
Q1	79.6 ± 0.3	79.5 ± 0.3
Q2	79.2 ± 0.3	79.2 ± 0.3
Q3	79.1 ± 0.2	79.1 ± 0.2
Q4	78.9 ± 0.3	79.0 ± 0.3
Q1 minus Q4	0.7 (-0.1, 1.5)	0.5 (-0.3, 1.4)
Slope of ln(adiponectin)*	0.45 (-0.19, 1.09)	-0.30 (-0.37, 0.96)
	**p for trend = 0.16**	**p for trend = 0.37**

Note 1: Model 1 was adjusted for race, sex, center, age, age^2^, amount of self-reported physical activity, body mass index (BMI) and smoking status (never, former, current) all at year 15 and height, height^2^at year 0. Model 2 was adjusted for all covariates in model 1 and for ln(insulin resistance in HOMA units) and ln(CRP) at year 15 examination.

Note 2: Quartile cutpoints for serum adiponectin concentrations are ≤6.99, 7.00-9.59, 9.60-14.39, and ≥ 14.40 mg/l. Mean ± standard deviation of ln(adiponectin) = 2.21 ± 0.62 mg/l

* Unit of slope was selected to match the adiponectin difference across Q1 to Q4. It was ml/(median level of adiponectin in quartile 1 (4.8 mg/l) - median level of adiponectin in quartile 4 (18.0 mg/l)).

### Year 15 serum adiponectin concentrations were not associated with a ten-year decline in lung function

Ten-year decline in lung function (FVC, FEV_1 _or FEV_1_/FVC) was not associated with year 15 serum adiponectin concentrations (Table 4). The rate of decline of lung function between years 10 to 20 were strikingly similar across all adiponectin quartiles (Table 4).

**Table 4:** Association between year 15 serum adiponectin concentrations and change in lung function over 10 years (year 20-year 10); CARDIA "calendar years".

*Year 15 adiponectin quartiles (Q)*	10 YEAR DECLINE IN LUNG FUNCTION PARAMETERS (95% CI)	
	MODEL 1 (n = 2,056)	MODEL 2 (n = 2,056)
	**FVC (ml) ± SE**	
Q1	-383 ± 14	-374 ± 14
Q2	-385 ± 14	-383 ± 14
Q3	-389 ± 13	-392 ± 13
Q4	-383 ± 14	-390 ± 14
Q1 minusQ4	0 (-42, 42)	16 (-27, 59)
Slope of ln(adiponectin)*	7 (-27, 41)	22 (-14, 57)
	**p for trend = 0.69**	**p for trend = 0.22**
		
	**FEV_1 _(ml) ± SE**	
Q1	-348 ± 11	-343 ± 11
Q2	-352 ± 12	-351 ± 12
Q3	-367 ± 11	-369 ± 11
Q4	-360 ± 12	-364 ± 12
Q1 minus Q4	12 (-22, 46)	21 (-14, 56)
Slope of ln(adiponectin)*	19 (-9, 47)	28 (-1, 56)
	**p for trend = 0.17**	**p for trend = 0.05**
		
	**FEV1/FVC (%) ± SE**	
Q1	-1.08 ± 0.17	-1.13 ± 0.18
Q2	-1.13 ± 0.18	-1.13 ± 0.18
Q3	-1.43 ± 0.17	-1.40 ± 0.17
Q4	-1.44 ± 0.18	-1.40 ± 0.18
Q1 minus Q4	0.36 (-0.17, 0.89)	0.27 (-0.27, 0.82)
Slope of ln(adiponectin)*	0.34 (-0.09, 0.77)	0.26 (-0.18, 0.71)
	**p for trend = 0.11**	**p for trend = 0.24**

Note 1: Model 1 was adjusted for race, sex, center, age, age^2^, amount of self-reported physical activity, body mass index (BMI) and smoking status (never, former, current) all at year 15 and height, height^2^at year 0. Model 2 was adjusted for all covariates in model 1 and for ln(insulin resistance in HOMA units) and ln(CRP) at year 15 examination.

Note 2: Quartile cutpoints for serum adiponectin concentrations are ≤6.99, 7.00-9.59, 9.60-14.39, and ≥ 14.40 mg/l. Mean ± standard deviation of ln(adiponectin) = 2.21 ± 0.62 mg/l

* Unit of slope was selected to match the adiponectin difference across Q1 to Q4. It was ml/(median level of adiponectin in quartile 1 (4.8 mg/l) - median level of adiponectin in quartile 4 (18.0 mg/l)).

### Year 15 serum adiponectin concentrations were positively associated with peak lung function in early adulthood

Peak FVC in early adulthood (*i.e*. the highest value among the years 0, 2 or 5 examinations) was 72 ml lower in the lowest *vs*. highest adiponectin quartile (4459 ml *vs*. 4531 ml; p for trend = 0.01). Similarly, peak FEV_1 _was 46 ml lower in the lowest vs. highest adiponectin quartile, although this difference did not reach statistical significance (p for trend = 0.07). After additional adjustment for insulin resistance and systemic inflammation at year 15, peak FVC and FEV_1 _were not associated with adiponectin (p for trend = 0.10 and 0.41 respectively). Peak FEV_1_/FVC was not associated with adiponectin concentrations. Decline in FVC or FEV_1 _from peak to 20 years was also not associated with adiponectin. Including lung function at year 10 in estimating peak lung function did not change the observed associations between peak lung function and adiponectin (data not shown).

In addition, there was no statistically significant interaction between serum adiponectin and sex in determining FVC, FEV_1 _or FEV_1_/FVC values at either years 10 or 20 examinations (p > 0.10). Analyses with logarithmically transformed adiponectin as a continuous variable showed results very similar to those observed with adiponectin analyzed as quartiles (tables 2, 3 and 4).

## Discussion

This study showed that serum adiponectin concentrations at the year 15 examination in the CARDIA study were positively associated with FVC and FEV_1 _values both at years 10 and 20 and these associations were independent of BMI. Serum adiponectin concentrations were however not associated with lung function decline between years 10-20. The associations between adiponectin and FVC and FEV_1 _at years 10 and 20 were no longer significant when additionally adjusted for insulin resistance and systemic inflammation.

Adiponectin is an anti-inflammatory adipokine. It inhibits pro-inflammatory cytokines such as tumor necrosis factor-alpha (TNF-α) and interleukin (IL)-6 and induces anti-inflammatory cytokines such as IL-10 and IL-1 receptor antagonist [20,21]. Adiponectin's insulin-sensitizing effect stimulates glucose utilization and fatty-acid oxidation [22,23]. A murine model has recently shown that genetically-induced adiponectin deficiency in the lungs of normal-weight mice maintained on a normal diet resulted in increased expression of TNF-α and matrix metalloproteinases (MMP-2 and MMP-12) in alveolar macrophages and "alveolar simplification and/or enlargement" due to abnormal postnatal alveolar development [15]. This murine study suggests that systemic adiponectin, independent of obesity, may have a protective effect on the lung through inhibition of alveolar macrophage-related inflammation.

This is the first population-based study to demonstrate that serum adiponectin is positively associated with lung function in humans. The difference in lung function across adiponectin quartiles was significant after adjusting for BMI or waist circumference, suggesting that it is not simply a function of global or abdominal adiposity. Based on current knowledge, it is likely that insulin resistance and systemic inflammation are mediators of the adiponectin-lung function association. However, the correlation between BMI, HOMA and CRP observed in this study makes it difficult to definitively classify these variables as confounders or mediators when evaluating the association between adiponectin and lung function. Hence, we present different scenarios with and without adjustment for these factors. Since serum adiponectin was associated with year 10 lung function but not subsequent decline in lung function, it is possible that the physiologic effect of serum adiponectin on lung function was established even before year 10 of the study and that classification by year 15 serum adiponectin concentrations might reflect lung growth abnormalities in early adulthood. The significant association observed between peak FVC in early adulthood and serum adiponectin concentrations at year 15 further supports this suggestion. Since serum adiponectin measurements were available about 10 years after measurement of peak lung function this finding needs to be confirmed in other longitudinal studies. The absence of adiponectin-lung function association after adjustment for HOMA and CRP suggests that the adiponectin effect on lung function might be explained by its anti-inflammatory and insulin sensitization effects.

Our results do not agree with two small previous cross-sectional studies of 31 and 15 patients with stable and established COPD that showed, without adjustment for obesity, no correlation between serum adiponectin concentrations and spirometric lung function [24,25]. The cross sectional design, presence of COPD, older age of subjects, confounding by BMI and limited power due to a small sample size are possible reasons for the observed discrepancy between these studies.

Usually, reduction in lung function with maintenance of FEV_1_/FVC ratio indicates restrictive ventilatory abnormality due to increased stiffness of the lungs or chest wall or reduced neuromuscular strength. Similar to observations made in mice models, inadequate alveolar morphogenesis may present with restrictive ventilatory abnormalities in humans. Another potential explanation includes the effect of adiponectin on peripheral or smaller airways (and associated premature airway closure and hyperinflation resulting in pseudo-restriction [26]). Similar small airway physiologic abnormalities, with thickened alveolar walls, have been previously described in patients with diabetes mellitus [27,28]. A recent study showed that phosphate buffered saline inhalation by adiponectin-deficient mice produced greater decrease in lung compliance compared to wild-type mice. The investigators suggested that this may be due to an increase in small airway closure from reduced lung elastic recoil [29]. Future studies with static lung volume estimation may help clarify the precise physiological changes associated with reduced serum adiponectin concentrations in humans.

The immediate clinical relevance of our finding of a modest association between serum adiponectin and lung function in young adults is unclear. It is nonetheless significant in the context of an epidemiological study because even modestly lower lung function early in life is associated with increased risk for both future lung [30] and cardiovascular diseases [31-33]. Since an inflammatory pulmonary milieu may increase the risk for adverse reactions to other occupational and environmental exposures manipulation of systemic adiponectin concentrations in early life (such as with diet, exercise, weight loss and medications) may have long-term effects on lung development, injury and remodeling.

The present study has several strengths, including the large number of generally healthy participants, inclusion of blacks and women, high quality spirometry data collected over a ten-year period, confirmation in a human population of findings demonstrated more mechanistically in a mouse-model, and excellent retention of the original cohort.

One limitation of the study is that the measurements of lung function (years 10 and 20) and serum adiponectin (year 15) did not coincide. As a result, it is not possible to establish temporality of associations definitively. Another limitation is the inability of the adiponectin assay to distinguish between its different multimeric forms. Thus, this study could not evaluate potential biological differences in multimeric forms of adiponectin on lung function. We have limited information on lung function values at very high adiponectin concentrations (adiponectin concentrations ≥30 mg/L, which was the 99^th ^percentile in our data). We reduced the influence of these high values by studying natural logarithmically transformed adiponectin and discuss this methodological issue further in the online supplement (Additional File 1). Finally, serum adiponectin concentrations may not reflect airway adiponectin concentrations.

In summary, this translational epidemiological study supports the hypothesis that lower serum adiponectin concentrations are associated with lower lung function in young adults. It further suggests that this association is independent of obesity and possibly mediated by insulin resistance and systemic inflammation. Longitudinal studies with long-term follow-up using non-invasive tests of peripheral airway function will help us evaluate the association between serum adiponectin concentrations in early adulthood and risk of lung and cardiovascular disease in later life.

## Abbreviations

BMI: Body mass index; CARDIA: Coronary Artery Risk Development in Young Adults; CRP: C-reactive protein; CV: Coefficient of variation; FVC: Forced vital capacity; FEV_1_: Forced expiratory volume in 1 second; HOMA: Homeostatic Model Assessment; IL: Interleukin; MMP: Matrix metalloproteinases; TNF-α: Tumor necrosis factor-alpha.

## Competing interests

RK has served as a paid consultant to AstraZeneca, Boehringer-Ingelheim, Dey Pharmaceuticals and Takeda Pharmaceuticals. He Serves on speakers' bureaus for AstraZeneca, Boehringer-Ingelheim, Pfizer and GlaxoSmithKline, and is the recipient of a research grant from GlaxoSmithKline. All other authors do not have any conflicts of interest to declare.

## Authors' contributions

BT, DRJ and AS conceived the research question. BT performed all statistical analyses and wrote the manuscript. DRJ directed writing and analysis. LJS, RK, and MDG participated in data interpretation and provided critical review of the manuscript. AS directed data analysis and worked closely with BT in writing the manuscript and provided input in interpretation of the data. The manuscript was reviewed and approved by the CARDIA Steering Committee. All authors have read and approved the final manuscript.

## Supplementary Material

Additional file 1 **Influence of the rare high adiponectin values: representing the predictor (adiponectin) as untransformed vs. log transform of adiponectin**. This file provides a detailed description of the distribution of adiponectin concentrations in the CARDIA study and describes the rationale for analyzing adiponectin as a log transformed variable (when analyzed as a continuous variable). Click here for file
