# Supplementary material for: Serum adiponectin is positively associated with lung function in young adults, independent of obesity: The CARDIA study
Source: Respir Res. 2010 Dec 9;11(1):176. doi: 10.1186/1465-9921-11-176 (PMC3014903; doi:10.1186/1465-9921-11-176)
Supplement: Additional file 1 — Influence of the rare high adiponectin values: representing the predictor (adiponectin) as untransformed vs. log transform of adiponectin. This file provides a detailed description of the distribution of adiponectin concentrations in the CARDIA study and describes the rationale for analyzing adiponectin as a log transformed variable (when analyzed as a continuous variable). [file 1465-9921-11-176-S1.DOCX]

**Influence of the rare high adiponectin values: representing the predictor (adiponectin) as untransformed vs. log transform of adiponectin.**

***Distribution of adiponectin concentrations in the CARDIA study***

Supplemental figure 1 shows the skewed distribution of adiponectin. There are very few participants (n=20) with adiponection concentrations >30 mg/l

**Figure 1: Distribution of adiponectin concentrations in the CARDIA study**

Supplemental figure 2 shows the distribution of adiponectin after log transformation.

**Supplemental figure 2: Distribution of log transformed adiponectin in the CARDIA study**

As shown in these figures the distribution of adiponectin is more normally distributed after log transformation.

***Association between untransformed adiponectin concentrations, log transformed adiponectin concentrations and year 10 lung function***

As shown in Supplemental table 1, the association between the log transformed adiponectin concentrations and year 10 lung function is similar (both magnitude of association and statistical significance) to that observed with adiponectin quartiles. However, the association between untransformed adiponectin and year 10 lung function is significantly different as compared to the other two analyses.

**Supplemental table 1: Association between year 15 serum adiponectin concentrations and year 10 lung function; CARDIA “calendar years”.**

| **Year 15 adiponectin concentrations** | **Year 10 FVC** | **Year 10 FEV_1_** | **Year 10 FEV_1_/FVC** |
| --- | --- | --- | --- |
| Adiponectin quartiles (Q1-Q4) | -81 (-153, -9);  p for trend = 0.005 | -50 (-109, 9);  p for trend = 0.01 | 0.11 (-0.51, 0.73);  p for trend = 0.73 |
| Slope of ln(adiponectin)* | -82 (-140, -23); p = 0.005 | -61 (-109, -13); p = 0.01 | 0.11 (-0.51, 0.73); p = 0.73 |
| Slope of adiponectin | -36 (-92 – 21); p = 0.20 | -26 (-72 – 20); p = 0.26 | 0.07 (-0.52 – 0.67); p = 0.81 |

Note 1: All models are adjusted for race, sex, center, age, age^2^, amount of self-reported physical activity, body mass index (BMI) and smoking status (never, former, current) all at year 15 and height, height^2^ at year 0.

Note 2: Quartile cutpoints for serum adiponectin concentrations are ≤6.99, 7.00-9.59, 9.60-14.39, and ≥ 14.40 mg/l.

* Unit of slope was selected to match the Q1 to Q4 quartile difference. It is ml/(median level of adiponectin in quartile 1 (4.8mg/l) – median level of adiponectin in quartile 4 (18.0 mg/l)).

The linear fit (adiponectin as a continuous variable) and the quartile fit (adiponectin as a categorical variable) are complementary.  The quartile fit is a check of the goodness of fit of the line, and the line is a summary of the linear tendency across quartiles. The analyses of adiponectin as a categorical variable represents a very easily understandable approximation to a spline fit (a fit with very little restriction on shape). Hence it is desirable for the linear fit to be in accordance with the quartile findings. Since there was a discrepancy in the results between the quartile fit and the untransformed adiponectin we reasoned that bringing the extreme high values closer together in this skewed distribution may affect the findings in this paper by reducing the influence of the very high adiponectin concentrations where we have relatively little data. To test this hypothesis, we deleted 20 observations where the adiponectin values were > 30 mg/L (99^th^ percentile of adiponectin distribution).

Supplemental table 2 shows that after deleting these 20 values, the analyses of adiponectin without transformation is very similar to the adiponectin analyses with log transformation.

**Supplemental table 2: Influence of high values of adiponectin on adiponectin –lung function association.**

| **Year 15 adiponectin concentrations** | **Year 10 FVC** | **Year 10 FEV_1_** | **Year 10 FEV_1_/FVC** |
| --- | --- | --- | --- |
| Slope of adiponectin  (n=2056) | -36 (-92 – 21); p = 0.20 | -26 (-72 – 20); p = 0.26 | 0.07 (-0.52 – 0.67); p = 0.81 |
| Slope of adiponectin after deleting values > 30 mg/L  (n=2036) | -65 (-154 - -9); p =0.03 | -45 (-96 – 6); p = 0.08 | 0.16 (-0.49 – 0.82); p = 0.63 |
| Slope of ln(adiponectin)*  (n=2056) | -82 (-140, -23); p = 0.005 | -61 (-109, -13); p = 0.01 | 0.11 (-0.51, 0.73); p = 0.73 |

Note 1: All models are adjusted for race, sex, center, age, age^2^, amount of self-reported physical activity, body mass index (BMI) and smoking status (never, former, current) all at year 15 and height, height^2^ at year 0.

Note 2: Quartile cutpoints for serum adiponectin concentrations are ≤6.99, 7.00-9.59, 9.60-14.39, and ≥ 14.40 mg/l.

* Unit of slope = ml/(median level of adiponectin in quartile 1 (4.8mg/l) – median level of adiponectin in quartile 4 (18.0 mg/l)).

This suggests that the extreme adiponectin values are strongly influencing the adiponectin-lung function association and hence log transformation of adiponectin is necessary. We have insufficient data regarding association between lung function and very high levels of adiponectin. The analyses between adiponectin (untransformed vs. log transformation), year 20 lung function and change in lung function showed a similar pattern to those presented here (data not shown).
